# Anxiety and depressive symptoms among COVID-19 patients in Jianghan Fangcang Shelter Hospital in Wuhan, China

**DOI:** 10.1371/journal.pone.0238416

**Published:** 2020-08-28

**Authors:** Ling-Ling Dai, Xi Wang, Tian-Ci Jiang, Peng-Fei Li, Yu Wang, Shu-Jun Wu, Liu-Qun Jia, Meng Liu, Lin An, Zhe Cheng

**Affiliations:** 1 Department of Respiratory and Critical Care Medicine, The First Affiliated Hospital of Zhengzhou University, Zhengzhou, Henan, China; 2 Team of Henan National Emergency Medical Rescue, Zhengzhou, China; Qazvin University of Medical Sciences, ISLAMIC REPUBLIC OF IRAN

## Abstract

Fangcang shelter hospitals were established in China during the coronavirus disease 2019 (COVID-19) pandemic as a countermeasure to stop the spread of the disease. To our knowledge, no research has been conducted on mental health problems among patients in Fangcang shelter hospitals. This study aimed to determine the prevalence and major influencing factors of anxiety and depressive symptoms among COVID-19 patients admitted to Fangcang shelter hospitals. From February 23, 2020, to February 26, 2020, we obtained sociodemographic and clinical characteristics information of COVID-19 patients in Jianghan Fangcang Shelter Hospital (Wuhan, China) and assessed their mental health status and sleep quality. Data were obtained with an online questionnaire. The questionnaire consisted of a set of items on demographic characteristics, a set of items on clinical characteristics, the Self-Rating Anxiety Scale, Self-Rating Depression Scale, and Pittsburgh Sleep Quality Index. Three hundred seven COVID-19 patients who were admitted to Jianghan Fangcang Shelter Hospital participated in this study. The prevalence of anxiety and depressive symptoms were 18.6% and 13.4%, respectively. Poor sleep quality and having ≥ two current physical symptoms were independent risk factors for anxiety symptoms. Female sex, having a family member with confirmed COVID-19, and having ≥ two current physical symptoms were independent risk factors for depressive symptoms. Anxiety and depressive symptoms were found to be common among COVID-19 patients in Fangcang Shelter Hospital, with some patients being at high risk.

## 1. Introduction

Originating as a cluster of unexplained cases of pneumonia, coronavirus disease 2019 (COVID-19) was first identified in Wuhan, Hubei Province, China in December 2019 [1]. Spreading rapidly worldwide, the COVID-19 pandemic is a global health threat with devastating consequences that can potentially impact the citizens of all nations [2].

Widespread outbreaks of infectious diseases, such as Ebola virus disease and severe acute respiratory syndrome, are not only associated with physical illness but also with psychological distress and symptoms of mental illness [3, 4]. Results from prospective studies have consistently suggested that psychological distress is a predictor of future health and disease outcomes [5]. As with other infectious diseases, preliminary evidence suggests that COVID-19 also causes public panic and mental health stress; symptoms of anxiety and depression are common psychological reactions to the COVID-19 pandemic, and may be associated with sociodemographic factors and sleep quality [6–9]. However, previous studies have focused mainly on COVID-19-related mental health issues among the general population, medical staff, children, pregnant women with their husbands, people with mental illness and individuals in self-isolation [10–13]. We know very little about the psychological effects of the disease on patients with COVID-19 [14].

To control the spread of infection and save lives, China has implemented COVID-19 countermeasures, including the establishment of Fangcang shelter hospitals in Hubei Province [15]. Fangcang shelter hospitals are a novel public health concept. These hospitals were established in China to assist in the country’s management of COVID-19 [16]. The Fangcang shelter hospitals are large, temporary hospitals built by converting public venues, such as stadiums and exhibition centers, into healthcare facilities to receive non-seriously ill individuals with positive SARS-CoV-2 tests from their families and communities, while providing disease monitoring, medical care, food, shelter, and social activities [16,17]. Fangcang shelter hospitals have been crucial to the quick containment of COVID-19 in China, relieving some of the enormous pressure on the healthcare system, and providing an encouraging example for other countries [18]. As the COVID-19 pandemic spreads globally, some countries, such as Serbia, have also built Fangcang shelter hospitals. Moreover, other countries, such as Iran, the United States, the United Kingdom, and Spain, have implemented similar measures [16].

Based on the evidence from previous research on the general population and on medical staff, we speculate that the mental health of the Fangcang shelter hospital patients is affected by the COVID-19 pandemic. To our knowledge, no previous studies have been conducted on the mental health of Fangcang shelter hospital patients during the COVID-19 pandemic. A better understanding of the psychosocial problems of Fangcang shelter hospital patients can provide important guidance in carrying out timely psychological interventions for targeted populations in need and in the management of the Fangcang shelter hospitals in any future outbreaks. Therefore, this study aimed to determine the prevalence and major influencing factors of anxiety and depressive symptoms among COVID-19 patients admitted to Fangcang shelter hospitals.

## 2. Methods

### 2.1 Design, setting, and participants

This was a cross-sectional study performed via an anonymous online questionnaire from February 23, 2020, to February 26, 2020, in Jianghan Fangcang Shelter Hospital in Wuhan, China. The study was designed and conducted by doctors who worked at the hospital, and was approved by the ethics committees of the First Affiliated Hospital of Zhengzhou University (no.2020-KY-169). All study respondents were made aware that participation in the study was voluntary and provided prior written informed consent online.

The inclusion criterion was a COVID-19 diagnosis based on the guidelines of the National Health Commission of the People’s Republic of China [19]. Exclusion criteria included a previously diagnosed severe psychiatric illness (e.g., schizophrenia, bipolar disorder, anxiety disorder, or depression disorder), inability to complete or failure to complete the online questionnaire, currently taking oral medication for a chronic disease (e.g., metoprolol, reserpine, prednisone, and methylprednisolone) that can cause side effects associated with anxiety, depression, and insomnia [20–22].

### 2.2 Measures

The participants scanned the quick response codes with their mobile phones and completed the questionnaires. The study questionnaire consisted of five main components: a set of items on demographic characteristics, a set of items on clinical characteristics, the Self-Rating Anxiety Scale (SAS), the Self-Rating Depression Scale (SDS), and the Pittsburgh Sleep Quality Index (PSQI).

Using the questionnaire, we collected data on the physical symptoms and comorbidities that had been discussed in previous literature [23]. Participants were asked to indicate whether they were currently experiencing any of the 14 listed physical symptoms (fever, coughing, sputum production, shortness of breath, chest pain, fatigue, soreness or discomfort in the throat, nasal congestion, conjunctival congestion, hemoptysis, headache, diarrhea, abdominal pain, myalgia, and arthralgia) or had any of the 9 listed comorbidities (chronic bronchitis or chronic obstructive pulmonary disease, asthma, hypertension, diabetes, coronary heart disease, cerebrovascular disease, connective tissue diseases, chronic renal disease, and cancer).

Anxiety and depressive symptoms were assessed using the Chinese versions of the SAS and SDS. The Chinese versions of the SAS and SDS have been proven to be effective instruments and have good psychometric properties when used in China (Cronbach's *α* coefficient was 0.82 and, 0.83 respectively) [24,25]. The SAS and SDS both contain 20 items, with responses based on a 4-point scale. For both scales, each question is based on mood experiences in the previous 7 days. An aggregate score of 20 is multiplied by 1.25, with higher scores indicating more severe levels of anxiety and depression [26]. Cutoff scores of ≥50 in SAS and ≥53 in SDS represent a positive screen for depression and anxiety symptoms [26].

Sleep quality was assessed using the Chinese version of the PSQI. The Chinese version of the PSQI has adequate reliability, with an internal consistency Cronbach’s α of 0.77 to 0.84 [27]. The PSQI consists of seven domains: sleep quality, sleep duration, sleep latency, habitual sleep efficiency, sleep disturbance, use of sleeping medications, and daytime dysfunction [28]. Responses to each item are based on a 3-point scale, with the total score ranging from 0 to 21. Higher scores indicate lower sleep quality. Poor sleep quality was defined as a total score ≥6 [29].

### 2.3 Statistical analysis

All statistical analyses were performed using IBM SPSS version 25 (IBM Corporation, Armonk, NY, USA). Continuous data that were normally distributed were presented as mean ± standard deviation, non-normally distributed data were described as median and first, third quartile: M (Q1, Q3); t-tests were used to compare the normal distribution of continuous data. Univariate analyses of anxiety and depression symptoms were performed using the chi-squared (*χ*^2^) test. Covariates with *P* <0.10 in the univariate analyses were included in the multivariate analyses. The multivariable logistic regression models were built using the forward LR variable selection method to identify independent factors associated with anxiety and depression. A *P*-value <0.05 was considered statistically significant.

## 3. Results

### 3.1 General characteristics and prevalence of anxiety and depressive symptoms

**Fig 1:**
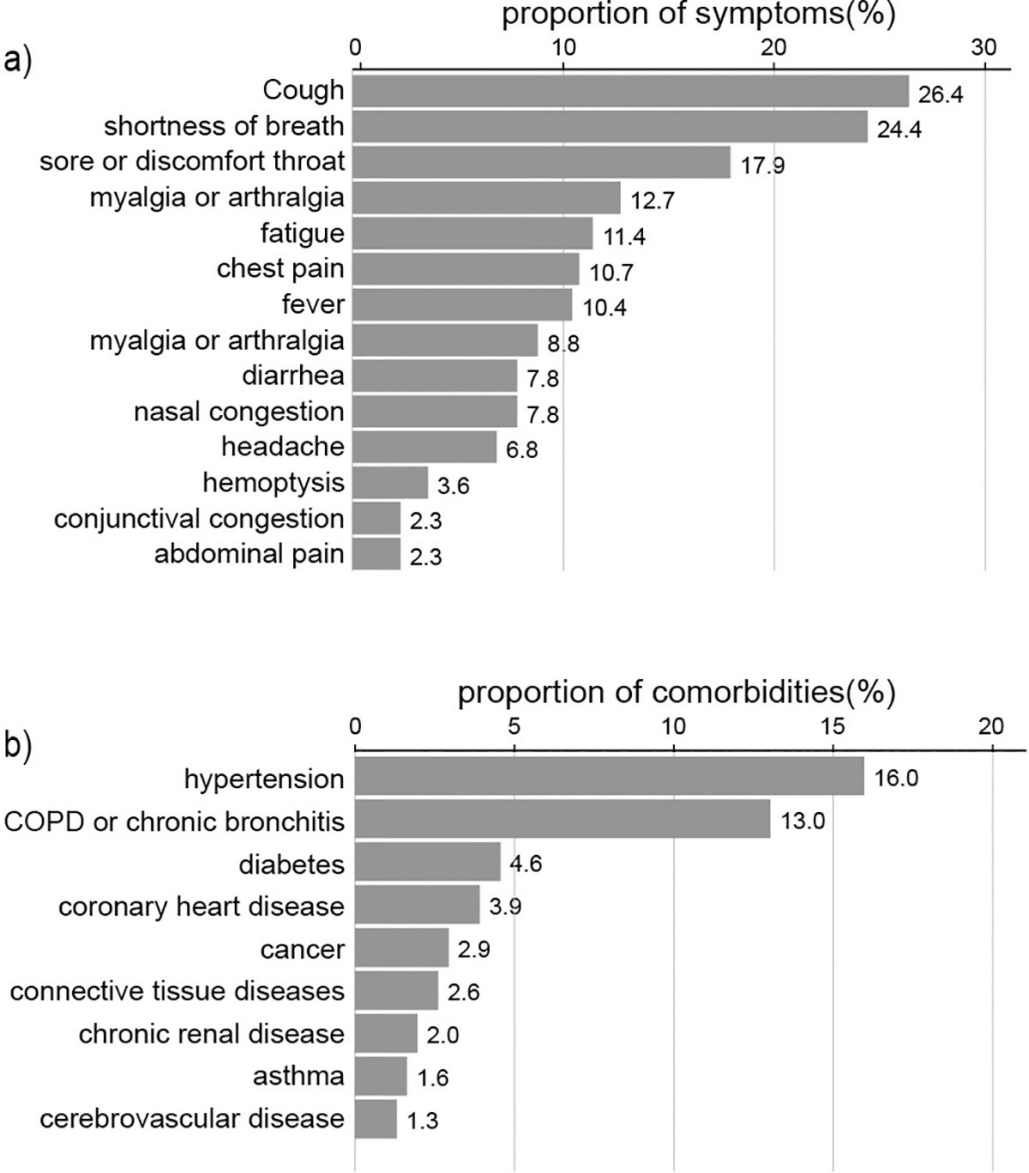
Histogram of the frequency distribution of comorbidities and current physical symptoms. a: Histogram of the frequency distribution of comorbidities of patients in Jianghan Fangcang Shelter Hospital in Wuhan, China. b: Histogram of the frequency distribution of current physical symptoms of patients in Jianghan Fangcang Shelter Hospital in Wuhan, China. COPD: Chronic obstructive pulmonary disease.

A total of 307 patients participated in the study. Among them, 57 (18.6%) experienced anxiety symptoms, and 41 (13.4%) experienced depressive symptoms. Two hundred sixty (84.7%) had poor sleep quality, as determined with the PSQI. The three most common coexisting illnesses were hypertension (16.0%), chronic bronchitis or chronic obstructive pulmonary disease (13.0%), and diabetes (4.6%), as shown in Fig 1A. There were 20 currently asymptomatic patients. The three most common current physical symptoms were coughing (26.4%), shortness of breath (24.4%), and soreness or discomfort in the throat (17.9%) (see Fig 1B).

Data on the demographic and clinical characteristics of the patients, and on the differences in incidence of anxiety and depressive symptoms among different groups are shown in Table 1. Based on covariates with *P* < 0.10 as screening covariates, having a family member who has been confirmed with COVID-19, number of current physical symptoms, symptoms change after hospitalization, and poor sleep quality were the related factors of anxiety symptoms, and gender, education level, smoking history, drinking history, having a family member who has been confirmed COVID-19, number of current physical symptoms, symptoms change after hospitalization, and poor sleep quality were the related factors of depressive symptoms (see Table 1).

**Table 1:** General characteristics of the sample.

Characteristic	Anxiety Symptoms	No Anxiety Symptoms	*χ*^2^	*p*	Depressive Symptoms	No Depressive Symptoms	*χ*^2^	*p*
Gender			1.627	0.202			17.169	<0.001
Male	28	146			11	163		
Female	29	104			30	103		
Age (year)							0.799	0.671
≤44	28	128			20	136		
45–59	25	94	1.288	0.525	18	101		
≥60	4	28			3	29		
Marital status			2.762	0.251			3.002	0.238
Single	5	32			5	32		
Married	46	205			31	220		
Divorced or Widowed	6	13			5	14		
Education level			0.074	0.964			5.264	0.072
Middle school or below	14	58			13	59		
High school	14	65			14	65		
College or above	29	127			14	142		
BMI (kg/m2)			0.274	0.872			0.869	0.647
<24	25	119			22	122		
24–28	24	97			14	107		
>28	8	34			5	37		
Comorbidity			0.993	0.319			0.021	0.885
Yes	29	109			18	120		
No	28	141			23	146		
Smoking history			0.752	0.386			4.039	0.044
Yes	10	57			4	63		
No	47	193			37	203		
Drinking history			1.099	0.294			3.024	0.082
Yes	10	60			5	65		
No	47	190			36	201		
Inpatient days			0.070	0.966			1.424	0.491
≤7	7	34			5	36		
8–14	9	39			4	44		
>14	41	177			32	186		
Family member confirmed COVID-19			3.334	0.068			4.707	0.030
Yes	32	107			25	114		
No	25	143			16	152		
Current nucleic acid result			2.010	0.156			0.747	0.388
Negative	36	132			25	143		
Positive	21	118			16	123		
Number of current physical symptoms			18.760	<0.001			14.812	<0.001
≤1	22	173			15	180		
≥2	35	77			26	86		
Symptoms change after hospitalization			5.952	0.051			4.696	0.096
Better	46	191			35	202		
Worse	5	8			3	10		
Unchanged	6	51			3	54		
Poor sleep quality			5.449	0.020			3.971	0.046
Yes	54	206			39	221		
No	3	44			2	45		

BMI, body mass index; chi-square test for categorical variables

### 3.2 Risk factors for anxiety and depressive symptoms

Results of the multivariate logistic regression analyses to determine the risk factors for anxiety and depressive symptoms are presented in Table 2. Poor sleep quality (odds ratio [OR], 3.655, 95% confidence interval [CI], 1.074–12.433; *P* = 0.038) and having ≥ two current physical symptoms (OR, 3.504; 95% CI, 1.919–6.398; *P* < 0.01) were independent risk factors for anxiety symptoms. The Omnibus Test of Model Coefficients showed that the model was significant, (*χ*^2^ = 23.905, *P* < 0.001). The Hosmer and Lemeshow test showed that the model had perfect goodness of fit (*χ*^2^ = 0.118, *P* = 0.943; see Table 2). Female sex (OR, 5.878; 95% CI, 2.657–13.005; *P* < 0.001), having a family member with confirmed COVID-19 (OR, 2.81; 95% CI, 1.337–5.911; *P* = 0.006), having ≥ two current physical symptoms (OR, 4.145; 95% CI, 1.994–8.616; *P* < 0.001) were independent risk factors for depressive symptoms. The Omnibus Test of Model Coefficients showed that model was significant, (*χ*^2^ = 40.508, *P* < 0.001). The Hosmer and Lemeshow test showed that the model had perfect goodness of fit (*χ*^2^ = 4.344, *P* = 0.630, see Table 2).

**Table 2:** Multivariate logistic regression analysis of factors influencing anxiety and depressive symptoms.

Variable	*B*	*SE*	*Walds*	*P*	*OR (95% CI)*
Models for anxiety symptoms					
Poor sleep quality (yes/no)	1.296	0.625	4.304	0.038	3.655 (1.074 to 12.433)
Number of current physical symptoms (≥2 vs ≤1)	1.254	0.307	16.657	<0.001	3.504(1.919 to 6.398)
Models for depressive symptoms					
Gender (female vs male)	1.771	0.405	19.111	<0.001	5.878(2.657 to 13.005)
Family member confirmed with COVID-19 (yes/no)	1.034	0.379	7.431	0.006	2.811(1.337 to 5.911)
Number of current physical symptoms (≥2 vs ≤1)	1.422	0.373	14.506	<0.001	4.145(1.994 to 8.616)

B, Partial regression weight, SE, Standard error, OR, odds ratio; CI, confidence interval; multivariate logistic regression analyses with forward stepwise variable selection

## 4. Discussion

This cross-sectional study examined the prevalence of anxiety, depression, and poor sleep quality among 307 patients in Jianghan Fangcang Shelter Hospital in Wuhan, China, 2 months after the start of the COVID-19 pandemic. Of all the participants, 18.57%, 13.36%, and 84.69% had anxiety symptoms, depressive symptoms, and poor sleep quality, respectively. In addition, using one-sample-tests, it was determined that both SAS (42.92±7.30) and SDS (39.77±10.11) scores of the participants of our study were higher than Chinese norms (SAS, 29.78±10.07, n = 1158; SDS, 33.46±8.55, n = 1340) (both P<0.001) [26], indicating more severe levels of anxiety and depressive symptoms among COVID-19 patients admitted to Fangcang hospitals, compared with the general public. Clearly, anxiety and depressive symptoms were common responses to the COVID-19 outbreak, and patients in the Jianghan Fangcang Shelter Hospital had severe levels of anxiety and depressive symptoms. The reason for this may be related to many factors: differing viewpoints on mask wearing, misperceptions in society, shortage of personal protective equipment, uncertainty about the progression of the pandemic, and fear of a difficult recovery from the disease [30–33].

In one previous Chinese study conducted in the initial stage of the pandemic, which reported a high prevalence of moderate to severe depressive symptoms, anxiety symptoms were found to be 16.5% and 28.8% among the general population, respectively [8]. In another Chinese study, the prevalence of anxiety and depressive symptoms among healthcare workers treating patients with COVID-19 was 44.6% and 50.4%, respectively [34]. This is in sharp contrast to the low prevalence of anxiety and depressive symptoms found in the present study. However, one Chinese study conducted within the same study period as that of the present study reported prevalence rates of anxiety and depressive symptoms among all the participants (including medical health workers and nonmedical health workers) of 10.4% and 10.6%, respectively [35]. Therefore, differences in prevalence rates may be due to differences in study periods. With the COVID-19 pandemic, the government of China has provided appropriate information and knowledge in a timely manner. Transparency and open communication can efficiently lower fear, anxiety, stigmatization, and discrimination [36]. Moreover, the National Health Commission of China has performed psychological crisis intervention through the general deployment of disease prevention and mental health professionals and expert groups providing psychological intervention for different subpopulations, including patient isolation in Fangcang shelter hospitals [16, 37]. Early psychological crisis intervention has reduced the prevalence of negative psychological outcomes caused by the COVID-19 outbreak.

Results of the multivariate logistic regression analyses indicated that poor sleep quality and having more current physical symptoms were risk factors for anxiety symptoms among patients in Fangcang shelter hospitals. Sleep provides time for the recuperation and rejuvenation of the brain. A substantial body of literature has shown that stressful life events and outbreaks of infectious diseases, including COVID-19, can affect sleep quality [34, 38–41], and 84.69% of the participants in the present study had poor sleep quality. Syntheses of longitudinal studies suggested that sleep quality was bidirectionally related to anxiety [42]. There is a large amount of data on the effects of sleep quality on anxiety symptoms in other populations, such as shift workers, firefighters, paramedics, pregnant females, and older adults. Poor sleep quality was found to be associated with higher risk for anxiety symptoms, and greater anxiety was found to be associated with poorer sleep quality [43–47]. Similarly, anxiety affects sleep quality because anxious people find it hard to fall asleep and wake up frequently [42]. In addition, the present study demonstrated that patients with more physical symptoms of COVID-19 were more vulnerable to anxiety symptoms. possible reasons are as follows: First, common symptoms of COVID-19, such as fever, shortness of breath, and headache can induce anxiety symptoms [48]. Second, patients with more symptoms are generally more serious than asymptomatic patients, and the prevalence of anxiety is also related to the severity of the disease [49,50]. Last, patients with more symptoms are more concerned about the progression of the illness.

Another finding from the present study was that females, patients with family member with confirmed COVID-19, and patients with more current physical symptoms were more likely to have anxiety symptoms. As early as the 1970s, Weissman underscored the gender differences in depression, and noted that females were more likely to experience depression than males [51]. Since then, there has been a proliferation of research and theories on gender differences in depression. One recent meta-analysis showed that females are more vulnerable to depression disorders and depression symptoms [52]. There is now a consensus that gender differences in depression have a multifactorial etiology; for example, there is a confluence of hormonal and neurodevelopmental changes that vary by sex during the pubertal transition and may influence gender differences in depression [52].

In addition, patients with family members diagnosed with COVID-19 were more vulnerable to depressive symptoms, owing to greater family burden and psychological distress [53, 54]. Compared with patients with less physical symptoms, patients with more physical symptoms were more likely to have depressive symptoms because they were more severe and the prevalence of depressive symptoms in relation to the severity of the disease [50].

This study had some limitations. First, this was a cross-sectional study, conducted within a short time frame. Therefore, future longitudinal studies are needed for follow-up and intervention. Second, psychological assessment was based on an online survey with self-report tools that had not been specifically designed for use with COVID-19 patients [55–57]. The use of clinical interviews and instruments designed specifically for COVID-19 patients is encouraged in future studies, in order to produce more comprehensive findings. Third, this was not a multinational, multicenter study, and therefore, further research is needed to produce more data on anxiety and depressive symptoms among patients in Fangcang shelter hospitals.

## 5. Conclusion

This study identified the prevalence rates and risk factors of anxiety and depressive symptoms among patients in Fangcang shelter hospitals. Anxiety and depressive symptoms were found to be common among the COVID-19 patients in the hospitals. Those with more physical symptoms and poor sleep quality demonstrated more vulnerability to anxiety symptoms. Females, patients with family members who had been diagnosed with COVID-19, and patients with more current physical symptoms were more vulnerable to depressive symptoms. The poorer the sleep quality, the more serious the symptoms of anxiety and depression. Our findings can aid in the development of interventions to reduce the adverse psychological impact of the COVID-19 pandemic on patients in Fangcang shelter hospitals in the future.

## Supporting information

S1 Appendix Data of this research for the values behind the means, standard deviations and other measures reported. (XLSX)
